# Checkpoint Inhibitors as High-Grade Gliomas Treatment: State of the Art and Future Perspectives

**DOI:** 10.3390/jcm10071367

**Published:** 2021-03-26

**Authors:** Pasquale Persico, Elena Lorenzi, Angelo Dipasquale, Federico Pessina, Pierina Navarria, Letterio S. Politi, Armando Santoro, Matteo Simonelli

**Affiliations:** 1 Department of Biomedical Sciences, Humanitas University, Via Rita Levi Montalcini 4, 20090 Milan, Italy; pasquale.persico@hunimed.eu (P.P.); elena.lorenzi@humanitas.it (E.L.); angelo.dipasquale@humanitas.it (A.D.); federico.pessina@hunimed.eu (F.P.); letterio.politi@hunimed.eu (L.S.P.); armando.santoro@humanitas.it (A.S.); 2 Oncology Department, IRCCS Humanitas Research Hospital, via Manzoni 56, 20089 Milan, Italy; 3 Neurosurgery Department, IRCCS Humanitas Research Hospital, via Manzoni 56, 20089 Milan, Italy; 4 Radiotherapy Department, IRCCS Humanitas Research Hospital, via Manzoni 56, 20089 Milan, Italy; pierina.navarria@humanitas.it; 5 Neuroradiology Department, IRCCS Humanitas Research Hospital, via Manzoni 56, 20089 Milan, Italy

**Keywords:** checkpoint inhibitors, immunotherapy, glioma, glioblastoma, PD-1, PD-L1

## Abstract

Glioblastoma (GBM) is the most common and aggressive malignant brain tumor in adults. Despite significant efforts, no therapies have demonstrated valuable survival benefit beyond the current standard of care. Immune checkpoint inhibitors (ICI) have revolutionized the treatment landscape and improved patient survival in many advanced malignancies. Unfortunately, these clinical successes have not been replicated in the neuro-oncology field so far. This review summarizes the status of ICI investigation in high-grade gliomas, critically presenting the available data from preclinical models and clinical trials. Moreover, we explore new approaches to increase ICI efficacy, with a particular focus on combinatorial strategies, and the potential biomarkers to identify patients most likely to benefit from immune checkpoint blockade.

## 1. Introduction

Glioblastoma (GBM) is the most common and lethal primary brain tumor in adults, with <10% of newly diagnosed patients alive at five years, despite an aggressive multimodality treatment approach, based on maximal safe surgical resection, followed by radiotherapy (RT) and temozolomide (TMZ) [1]. Previous investigations of different strategies, such as intensification of chemotherapy (CT) [2], targeting dysregulated cell-signaling pathways [3], and antiangiogenic therapy [4,5], failed to improve survival in randomized clinical trials and the standard of care (SOC) remained unchanged over the last decade. No truly effective therapy exists at recurrence, and therefore, innovative therapeutic approaches are urgently needed [6]. In the last years, immunotherapy, with its own concept of boosting tumor-specific adaptive immunity instead of directly targeting cancer cells, has recently emerged as a cornerstone of modern oncology [7].

High-grade gliomas (HGG) tumor microenvironment (TME) is unique in its cellular composition and accessibility to immune cells, being insulated by the blood-brain-barrier (BBB). Tumor-associated macrophages (TAM) are highly prevalent in GBM, constituting up to 30% of TME, and tend to be pro-tumorigenic. Moreover, TAMs produce low pro-inflammatory cytokine levels, poorly express costimulatory molecules involved in T-cells activation and have a pleiotropic capability to suppress cluster differentiation 8 (CD8)+ T cell activity in GBM. Dendritic cells (DC) are professional antigen-presenting cells (APC) with a crucial role in initiating and shaping immune responses. Recent transcriptomic analyses of human GBM samples confirmed DCs’ presence, but their role in glioma is still a matter of debate. Neutrophils are the most prevalent immune population, comprising up to 70% of total leukocytes in the body. In glioma, they support progression, invasiveness, and angiogenesis through the secretion of elastase and metalloproteases. Neutrophils-to-lymphocytes ratio (NLR) in blood correlates with glioma grade and is highest in GBM. Moreover, an elevated NLR is associated with poor clinical outcomes in GBM patients [8,9].

HGG TME is characterized by low numbers of CD8+ cytotoxic infiltrating T cells (TIL). Conversely, CD4+ regulatory T cells (Tregs) levels are increased in GBM. Tregs exert an immune-suppressive role as they secrete transforming growth factor beta and interleukin 10 (IL-10), limiting IL-2 and interferon (IFN) production and suppressing T cells’ survival and activity. GBM actively recruits Tregs producing soluble factors, such as C-C motif chemokine ligand 2. Moreover, TAMs expressing T-cell immunoglobulin and mucin domain-containing molecule-4 and DCs producing indoleamine 2,3 dioxygenase (IDO) also play a direct role in Tregs induction. Lastly, natural killer (NK) cells are innate lymphoid cells representing about 10% of all circulating lymphocytes. In GBM, they represent a minor component of TME and are reduced in glioma patients’ bloodstream as compared to healthy controls [8,10].

In physiological conditions, a series of stimulatory and inhibitory pathways called “immune checkpoints” regulate and optimize immune responses’ strength and magnitude, limiting potential harmful effects on normal tissues [11]. Aberrant immune checkpoint signaling is one of the fundamental mechanisms used by tumor cells to dampen the immune response and avoid detection and killing. The upregulation of the programmed cell death 1(PD-1)/programmed death-ligand 1 (PD-L1) axis is one of the key contributors to immunosuppression in glioma TME. PD-1/PD-L1 signaling impairs T-cell activation, cytotoxicity, and proliferation and enhances Tregs survival. Moreover, glioma cells upregulate PD-L1 in myeloid cells and Tregs. Cytotoxic T-lymphocyte-associated protein 4 (CTLA-4) is expressed by naïve T cells and negatively interacts with APCs reducing early stages of T-cell expansion. CTLA-4 is also constitutively expressed on Tregs, being a key contributor to their immunosuppressive functions. Moreover, the upregulation of alternative immune checkpoints, as T-cell immunoglobulin and mucin-domain containing-3 (TIM-3) and lymphocyte-activation gene 3 (LAG-3), is a hallmark of GBM, leading to a severe exhausted T-cell phenotype [10,12]. Immunomodulating monoclonal antibodies (mAb) targeting these immune checkpoints can reverse T-cell dysfunctionality, enhancing immune control, and restoring antitumor activity [11].

Immune checkpoint inhibitors (ICI) have revolutionized the treatment paradigm of several historically resistant cancers, achieving regulatory approvals for several different malignancies and indications [13,14]. Since the Food and Drug Administration (FDA) approval of ipilimumab (anti-CTLA-4 mAb) in 2011, PD-1 inhibitors nivolumab, pembrolizumab, cemiplimab, and PD-L1 inhibitors atezolizumab, avelumab, and durvalumab have entered clinical use. These remarkable successes have raised great expectations in whether these agents could be such effective also in the context of neuro-oncology, and an intensive clinical investigation has been undertaken. Unfortunately, to date, these efforts have been entirely unsuccessful.

Here, we critically review the most relevant preclinical and clinical data on the use of immune checkpoint modulation as treatment of diffuse malignant gliomas available so far. We will further discuss the current landscape of biomarkers and potential novel insights to be explored to raise the bar beyond current disappointing results and make significant progress in this field.

## 2. Preclinical Data

Impressive and long-lasting remissions with checkpoint blockade have been observed in several orthotopic murine glioma models. These results provide a strong immunological rationale to support ICI clinical use and even the potential synergy with other anticancer treatment modalities, including RT and CT. CTLA-4 blockade conferred long-term survival in up to 80% of treated mice, restoring normal CD4+ T-cell count and proliferative capacity and inducing resistance to Tregs without directly affecting their suppressive functions [15]. The combination of PD-1 blockade and RT was significantly more effective than either modality alone (*p* < 0.05 by log-rank Mantle–Cox), with 15 to 40% of mice treated with the combination achieving long-term survival (>90 days after tumor implantation) [16]. After rechallenging with flank injections of mouse glioma 261-luciferase expressing (GL261-Luc) cells, no tumors developed in the survivor mice, testifying the induction of a long-lasting systemic immunologic memory [16].

Even the combination of PD-1 blockade and TMZ showed superior survival to either therapy alone (42 days in the combination group versus 28 days in the anti-PD1 group versus 30 days in the TMZ group; *p* < 0.01) [17]. In this study, the number of infiltrating CD4+ and CD8+ T-cells and tumor size reduction was greater in the combination arm [17]. An apparent synergy between anti-PD-1 therapy and TMZ was also observed by Park et al. in another orthotopic murine GBM model. Complete remissions occurred in 44.4% of mice (4/9) treated with anti-PD-1 monotherapy and in all mice (9/9) after combination therapy, whereas no effect was seen with TMZ alone [18]. Anti PD-1 monotherapy increased TILs, while TMZ, alone or in combination, reduced TILs [18]. The absence of tumor growth after rechallenge and the presence of antitumor memory T cells were observed only in the anti-PD-1 monotherapy group [18].

Preclinical experiences also demonstrated the potential effectiveness of co-targeting different immune checkpoints in GBM. In a study by Wainwright et al., the triple simultaneous blockade of CTLA-4, IDO, and PD-L1 led to a 100% of long-term survivor mice and a significant reduction in tumor-infiltrating Tregs levels [19]. In a syngeneic orthotopic murine glioma model, Kim et al. showed upregulation of TIM-3 expression in TILs and the accumulation of severely anergic PD-1+/TIM-3+ lymphocytes. Dual blockade of TIM-3 and PD-1 in combination with stereotactic radiosurgery (SRS) was associated with a 100% overall survival (OS) rate (*p* < 0.05) and rejection of tumor rechallenge [20]. The immune TME was favorably shaped, with an improved CD8+/Tregs ratio, upregulation of pro-inflammatory cytokines such as IFN-γ, tumor necrosis factor alpha, IL-17a, and a lower fraction of Tregs [20]. T cell immunoreceptor with immunoglobulin and ITIM domain (TIGIT) and PD-1 co-targeting led to a significant improvement in OS compared to single blockade alone (*p* = 0.0366 and <0.0001, respectively), and about half of the mice were long-term survivors (*p* < 0.0001) with dual therapy [21]. Clinical efficacy was correlated with increased T cell functionality and downregulation of suppressive Tregs and tumor-infiltrating DCs [21]. Readorn et al. tested the efficacy of CTLA-4, PD-1, PD-L1, and PD-ligand 2 (PD-L2) targeting in an orthotopic immunocompetent murine model. PD-L1 and CTLA-4 blockade was the most active approach, with 75% of long-term, tumor-free survivor mice even in the case of advanced or later-stage tumors [12]. Neither tumor persistence at histopathologic examination nor tumor recurrence after intracranial tumor rechallenge were detected in long-term surviving mice. Dual blockade of PD-L1 and CTLA-4 was associated with favorable changes in the immune TME, such as increased infiltration of CD8+ T cells, activation of NK cells, and reduction in immunosuppressive subpopulations, including Tregs, myeloid-derived suppressor cells, PD-1+ T cells, and exhausted PD-1+/ TIM-3+ T- cells [12].

One of the most significant issues in interpreting such preclinical data is that glioma animal models developed by implanting cancer cell lines in mice do not accurately reflect human GBM immunobiological complexity and therefore result poorly appropriate to investigate immune checkpoint modulation strategies. For example, the commonly used GL261 syngeneic murine model is moderately immunogenic and conversely to human gliomas expresses clonotypic, homogeneous, and robust levels of PD-L1. Despite limitations derived by cost, low engraftment rates, and slow tumor growth, the advent of humanized mice models with patient-specific tumor and immune system indeed represents a significant step forward. Humanized mice tumor models are generated by the engraftment of human tumor cell lines, cancer stem cells or patient-derived xenografts into immunodeficient mice with a human immune system. The human immune cells are reconstituted through the transplantation of peripheral blood mononuclear cells or hematopoietic stem cells. As a result, these models mimic human tumor and immune system interactions and offer a more realistic representation of immunotherapy safety and clinical response. Nevertheless, they remain unable to fully recapitulate the TME of human gliomas and glioma patients’ clinical features [22,23]. Thus, the current lack of reliable preclinical models to investigate the strength of the rationale behind immunotherapeutic approaches is undoubtedly one of the underlying causes of repeated clinical failures seen in the latest years.

## 3. Clinical Data

Given these encouraging preclinical results and durable responses observed in brain metastases from other cancer types, such as melanoma and lung carcinoma, an intensive clinical investigation has been undertaken to explore the potential effectiveness of immune checkpoint modulation as a treatment strategy for GBM patients.

Here we report clinical data available so far from early-phase to first randomized trials split in the adjuvant, recurrent, and neoadjuvant setting, with a particular focus on the most recent experiences not yet reviewed elsewhere. Results of the trials reported in this review are summarized in Table 1.

### 3.1. Adjuvant Setting

The safety of combining nivolumab with standard RT with or without TMZ in newly diagnosed GBM was firstly demonstrated in two cohorts (1c; 1d) of the prospective clinical trial Checkmate 143 (NCT02017717). Treatment-related adverse events (TRAE) occurred in 67% (1c) and 70% (1d) of patients, with the most common being fatigue (28%, 26%), headache (21%, 13%), and increased alanine transaminase (ALT) (16%, 9%). Grade 3–4 TRAEs occurring in at least two patients included increased ALT (5%; 6%) and lipase (2%; 8%) [24]. Other two small early-phase trials demonstrated the safety of adding PD-L1 blockade (atezolizumab and avelumab) to the SOC in newly diagnosed GBM. Promising, albeit preliminary signs of efficacy with no new safety alerts, were reported in both studies. In the phase I/II study of atezolizumab (*n* = 60, NCT03174197), median progression-free survival (PFS) and median OS were 10.6 months and 19 months, respectively [25]. A median PFS of 11.9 months and an overall response rate (ORR) of 50% were reported in the phase II study of avelumab (*n* = 24, NCT03047473) [26]. An open-label, multicenter phase II trial (NCT02336165) evaluated the safety and efficacy of durvalumab in five cohorts of GBM patients. Cohort A included 40 patients receiving durvalumab combined with standard RT, followed by durvalumab monotherapy without TMZ in O^6^-methylguanine-DNA methyltransferase (*MGMT*) unmethylated newly diagnosed patients. At a median follow-up of 24.5 months, median OS was 15.1 months (95% confidence interval [CI]: 12.0, 18.4)), OS at 12 months (OS-12) was 60% (90% CI: 46.1, 71.4) [27]. Grade ≥ 3 adverse events (AE) occurred in fourteen (35%) patients, the most common being asymptomatic increased lipase (*n* = 6) and increased amylase (*n* = 2) [27].

Two large, randomized trials testing the addition of nivolumab to first-line therapy in newly diagnosed GBM have already closed the accrual. The CheckMate 498 (NCT02617589) was an open-label phase III trial for unmethylated *MGMT* patients comparing the standard Stupp regimen versus nivolumab + RT. In May 2019, Bristol–Meyers–Squibb (BMS) announced that the CheckMate 498 trial did not meet its primary endpoint of OS at the final analysis [28]. CheckMate 548 (NCT02667587) was a single-blind phase II trial, comparing nivolumab + TMZ + RT versus placebo + TMZ + RT in patients with methylated *MGMT* promoter. The trial was later amended to become a randomized, triple-blind phase III trial with an increased sample size of approximately 693 subjects and both PFS and OS as primary endpoints. Recently, BMS reported that even the CheckMate 548 trial failed to meet PFS and OS co-primary endpoints in the intention-to-treat population, as well as in patients with no baseline corticosteroid use [29]. Waiting publication of final reports of both trials, along with sub-group and biomarkers analyses, these data certify the failure of the anti-PD-1/PD-L1 blockade in addition to SOC in newly diagnosed GBM.

### 3.2. Recurrent Setting—Monotherapy Trials

The first prospective clinical evaluation of ICI in recurrent GBM was represented by the previously mentioned multicohort phase I trial Checkmate 143. Cohorts 1 and 1b evaluated the safety, tolerability, and immunologic effects of nivolumab alone or combined with ipilimumab at two different dosing schedules in patients with recurrent GBM. In cohort 1, 20 patients were randomized 1:1 to either nivolumab monotherapy 3 mg/kg every 2 weeks (Q2W) (NIVO3, *n* = 10) or nivolumab 1 mg/kg + ipilimumab 3 mg/kg every 3 weeks (Q3W) for 4 doses, followed by nivolumab 3 mg/kg Q2W (NIVO1 + IPI3, *n* = 10). In the non-randomized cohort 1b, 20 patients received nivolumab 3 mg/kg + ipilimumab 1 mg/kg Q3W for 4 doses, followed by nivolumab 3 mg/kg Q2W (NIVO3 + IPI1). Nivolumab monotherapy was better tolerated than the combination of nivolumab + ipilimumab, as no grade 3/4 TRAEs were reported in the nivolumab monotherapy arm, whereas they occurred in nine (90%) and six (30%) patients, respectively, in NIVO1+IPI3 and NIVO3+IPI1 arm [30]. In total, three patients achieved a partial response (PR) (NIVO3, *n* = 1; NIVO3+IPI1, *n* = 2), and eight patients a stable disease (SD) for ≥12 weeks (NIVO3, *n* = 2; NIVO1+IPI3, *n* = 2; NIVO3+IPI1, *n* = 4), according to response assessment in neuro-oncology (RANO) criteria [30]. Median (95% CI) OS was 10.4 (4.1–22.8), 9.2 (3.9–12.7), and 7.3 (4.7–12.9) months in patients treated with NIVO3, NIVO1 + IPI3, and NIVO3 + IPI1, respectively [30]. Most patients (68%) had tumor-cell PD-L1 expression ≥ 1%. Immune-mediated effects mimicking radiographic progression occurred in two patients [30].

The KEYNOTE-028 trial (NCT02054806) was a multicohort phase Ib study designed to evaluate the safety and efficacy of pembrolizumab) in 20 advanced solid tumor types. The recurrent GBM cohort (*n* = 26) evaluated patients with bevacizumab-naïve recurrent GBM and a PD-L1 expression on >1% of stromal and tumor cells. Patients received pembrolizumab 10 mg/kg Q2W for a maximum of 24 months. Then, two PR, lasting 8.3 and 22.8 months, occurred. PFS (median, 2.8 months; 95% CI, 1.9–8.1 months) rate at six months (PFS-6) was 37.7%, and the OS (median, 13.1 months; 95% CI, 8.0–26.6 months) rate at 12 months was 58% [32]. A total of 19 patients (73%) experienced TRAEs, of whom five were severe (one arthritis, one colitis, one lymphopenia, one syncope, one type 2 diabetes mellitus) [32]. No correlation between the level of PD-L1 expression, gene expression profile score, or baseline steroid use and clinical benefit was found 32]. The safety and efficacy of the PD-L1 inhibitor atezolizumab were assessed in another phase Ia clinical trial (NCT01375842). Among 16 recurrent GBM patients, one had a PR, three patients experienced SD, and the median OS was 4.2 months (1.2–18.8) [33]. No grade 3–4 TRAEs were observed. A total of two patients with isocitrate dehydrogenase 1 (*IDH1*)-mutant tumors and one with a DNA polymerase epsilon (*POLE*)-mutant tumor reached a survival ≥ of 16 months [33]. Patients with low baseline levels of circulating CD4+ T cells and B cells (<0.40 × 10^6^ cells/ mL and <0.19 × 10^6^ cells/mL, respectively) showed a trend toward reduced PFS and OS [33]. In the previously mentioned multicohort phase II NCT02336165 trial, cohort B evaluated durvalumab as monotherapy in 31 recurrent bevacizumab-naïve GBM patients, showing PFS-6 of 20%, a 6-month OS rate (OS-6) of 59%, and an OS-12 of 44.4% [34]. Grade 3 AEs occurred in 9.7% of patients, while no grade 4 events were observed [34].

Preliminary results of the Checkmate 143 mentioned above led to the opening of a large randomized open-label phase III study (expansion of cohort 2) directly comparing the efficacy and safety of nivolumab monotherapy versus the anti-vascular endothelial growth factor A (VEGF-A) mAb bevacizumab in GBM patients at first recurrence after standard chemoradiation. The trial randomized 369 patients to receive either nivolumab 3 mg/kg Q2W (*n* = 184) or bevacizumab 10 mg/kg Q2W (*n* = 185) until investigator-assessed progressive disease (PD) or unacceptable toxicity. At a median follow-up of 9.5 months, no statistically significant differences in survival were observed, with a median OS of 9.8 months (95% CI, 8.2–11.8 months) in nivolumab arm versus 10 months (95% CI, 9.0–11.8 months) in bevacizumab arm (95% CI, 9.0–11.8 months); hazard ratio (HR) 1.04; 95% CI, 0.83–1.30; *p* = 0.76 [31]. Even ORR was in favor of bevacizumab (23.1%; 95% CI, 16.7–30.5%) versus 7.8% (95% CI, 4.1–13.3%), while the median duration of response (DOR) resulted significantly higher in patients treated with nivolumab (11.1 versus 5.3 months) [31]. Hypothesis-generating data from subgroup analyses showed that the corticosteroid use at baseline was unfavorably associated with outcomes in the nivolumab group (HR, 1.41; 95% CI, 1.01–1.97) [31]. In a multivariable analysis, no baseline corticosteroid use (HR, 0.59; 95% CI, 0.36–0.95) and methylated *MGMT* promoter status (HR, 0.47; 95% CI, 0.29–0.78) were each associated with longer OS in the nivolumab group [31]. Combining these two factors, patients with methylated *MGMT* promoter and no baseline corticosteroid use, treated with nivolumab, showed a trend toward longer OS (17.0 vs. 10.1 months; HR, 0.58; 95% CI, 0.30–1.11) [31]. All-grades TRAEs incidence was broadly similar in both arms (57% in the nivolumab group versus 58% in the bevacizumab group), with also similar rates of grade 3/4 TRAEs with nivolumab (18.1%) and bevacizumab (15.2%). The most common AEs were fatigue in the nivolumab group and hypertension in the bevacizumab group; no treatment-related deaths were reported [31].

Checkmate 143 is the first completed randomized trial of checkpoint blockade in recurrent GBM for which we have the definitive data available. Primary and secondary survival endpoints were not met, and indeed the study is to be considered entirely negative. These disappointing results can be explained at least in part by the extremely challenging setting of recurrent GBM characterized by a potent local and systemic immunosuppression frequently worsened by the use of steroids, hardly reversible by the sole anti-PD-1 blockade. Even the selection of bevacizumab with its well-known steroid-sparing effects as a competitor arm in the CheckMate 143 has been largely debated and should be considered when interpreting findings of this trial. On the other hand, what emerges just as clearly from this study is that a small subgroup of patients receiving anti-PD-1 therapy can obtain a durable disease control. *MGMT* promoter methylation and no corticosteroid use seem to characterize this subpopulation and will be tested as potential selection factors for future immunotherapy trials in this setting.

### 3.3. Recurrent Setting—Combination Trials

To improve these modest results and patients’ responsiveness, several clinical trials exploring the safety and efficacy of PD-1/PD-L1 blockade combined with other anticancer modalities such as antiangiogenic agents, radiation, or novel immunotherapeutics, have been undertaken.

#### 3.3.1. Checkpoint Blockade and Antiangiogenic Treatments

Among the most promising combination strategies, a robust immunological rationale supports ICIs’ combination with anti-VEGF treatments. First, the abnormal tumor vasculature acts as a physical barrier to effector immune cell infiltration, while hypoxia favors the recruitment of immunosuppressive cells into the TME. Moreover, VEGF, the primary driver of tumor angiogenesis, represents also a potent immunosuppressive factor active in both innate and adaptive immunity by inhibiting DCs maturation and antigen presentation, inducing CD8+ T-cells apoptosis, promoting Tregs activity, and polarizing TAMs towards a pro-tumoral phenotype [49]. Anti-VEGF therapy could help restore an immune-supportive TME in GBM, and thanks to its well-known vasculature-normalizing effects, to enhance the intratumoral immune cell infiltration [50].

The anti-VEGF mAb bevacizumab has been used in the management of recurrent GBM for over a decade [51] and represented the antiangiogenic agent primarily chosen for combination approaches with ICIs. Bevacizumab is also able to decrease the need for steroids for brain edema control, and this can be of great importance since steroid use is thought to be associated with decreased survival and appears to abrogate the efficacy of immunotherapies [51,52].

In the multicohort phase II NCT02336165 trial, the combination of PD-L1 blockade (durvalumab) and bevacizumab was explored in three distinct cohorts. In total, 66 recurrent bevacizumab-naïve GBM patients were enrolled in two-dose group (Cohort B2: durvalumab + bevacizumab 10 mg/kg Q2W; B3: durvalumab + bevacizumab 3 mg/kg Q2W) leading only to modest signs of activity (cohort B2 PFS-6: 15.2%; cohort B3 PFS-6: 21.1%) [36]. Grade 3–4 TRAEs were observed in 24.2% and 6.1% of patients in cohort B2 and B3, respectively [36]. In cohort C, 22 recurrent, bevacizumab refractory GBM patients received durvalumab + bevacizumab 10 mg/kg Q2W. At the data cut-off, eight patients (36%) had OS ≥ 22 weeks, eleven patients (50%) had PFS ≥ eight weeks, and three were still alive [37]. Common TRAEs were fatigue, increased ALT, constipation, and headache, mostly mild-to-moderate in severity [37].

The addition of bevacizumab to pembrolizumab was explored in a randomized phase II study for bevacizumab-naïve recurrent GBM patients (NCT02337491). In total, 50 patients were randomized to receive the combination (cohort A), while 30 patients were treated with pembrolizumab alone (cohort B). PFS-6 was 26.0% (95% CI: 16.3, 41.5) and 6.7% (95% CI: 1.7, 25.4) in the combination arm and in the pembrolizumab monotherapy arm, respectively [38]. Patients treated with the sole pembrolizumab had a better outcome (median OS 10.3 months, 95% CI: 8.5, 12.5) versus 8.8 months, 95% CI: 7.7, 14.2) [38]. Immune biomarkers, including PD-L1 expression, TIL infiltration, and immune activation gene expression profile, were not associated with OS [38]. Baseline dexamethasone use as well as lack of tumor *MGMT* methylation and *IDH1* mutation correlated with poorer OS. Analysis of plasma angiokines revealed that elevated baseline placental growth factor and soluble VEGF receptor-1 (cohort B) and post-therapy VEGF (cohort A) levels correlated with poorer survival [38].

In the same setting, the combination of axitinib (high-affinity tyrosine kinase inhibitor of the VEGF-receptors) and avelumab (NCT03291314) did not demonstrate a sufficient level of activity to deserve further investigation. PFS-6 was 22.2% (95% CI 6.5% to 37.9%) and 18.5% (95% CI 3.8% to 33.2%) in patients with a baseline use of steroid <8 mg and >8 mg of methylprednisolone, respectively [39]. AEs of special interest were pulmonary embolism (6%, all grade 4), immune-related hepatitis (4%; one grade 2, one grade 3), immune-related pneumonitis (2%; grade 3), psoriasiform rash (2%; grade 2), and microscopic colitis (2%, grade 2) [39].

#### 3.3.2. Checkpoint Blockade and Radiation Therapy

Accumulating evidence fostered the clinical evaluation of RT in combination with checkpoint blockade. Mainly, the use of SRS has gained popularity across multiple tumor types, owing to the potential to promote antigen release, limit the systemic immunosuppression associated with RT, and therefore assist in initiating an antitumor immune response. Radiation induces tumor cells’ immunogenic death, stimulating the release of tumor-associated antigens and increasing antigen presentation, promoting a pro-inflammatory TME, enhancing antitumor T-cells recruitment and antitumor responses [53]. The potential synergistic effects of RT and ICIs are also supported by previously described murine glioma models showing prolonged survival, increased TILs levels, and reduced local immunosuppression [16,20].

An open-label, single-arm phase I trial (NCT02313272) investigated the triple combination of pembrolizumab, hypofractionated stereotactic irradiation (HFSRT), and bevacizumab in 32 recurrent HGG patients (bevacizumab naïve, *n* = 24; bevacizumab resistant, *n* = 8). Treatment was well tolerated, with only one discontinuation due to grade 3 transaminases elevation. ORR in the bevacizumab naïve and bevacizumab resistant cohorts was 83% and 62.5%, respectively [40]. Median PFS was 7.92 and 6.54 months, and median OS was 13.45 and 9.3 months in bevacizumab naïve and bevacizumab resistant patients, respectively [40]. In a phase I/II clinical trial evaluating the combination of HFSRT with durvalumab for recurrent GBM (NCT02866747), one dose-limiting-toxicity (DLT) was reported among six patients (grade 3 immune-related vestibular neuritis). At the time of analysis (January 2019), all the patients had a PD, and four were still alive; interim analysis of the phase II part is ongoing [41]. A phase II, open-label, single-arm, multicenter study evaluated avelumab combined with HFSRT (NCT02968940) in adults with secondary *IDH*-mutant GBM previously treated with RT and CT. In the safety lead-in cohort of the study (*n* = 6), no DLT was observed. Median PFS was 4.2 months (range 1.4 to 5.7), median OS was 10.1 months (range 6.8 to 21) [42]. After this analysis, the study closed due to slow accrual. Finally, a phase II study evaluated nivolumab in combination with HFSRT and bevacizumab in recurrent *MGMT* methylated GBM (NCT03743662). As recently presented at Society for Neuro-Oncology (SNO) 2020 conference, at a median follow-up of 8.7 months, median PFS and OS were, respectively, 6.7 and 16 months, with a one-year OS of 59% [43]. Treatment was well-tolerated, with only one ≥ grade 3 adverse event (amylase increase) [43].

#### 3.3.3. Co-Targeting of Different Immune Checkpoints

The simultaneous modulation of different immune checkpoints or the targeting of different immune cell populations (e.g., TILs and TAMs) is another intriguing combination strategy currently under intensive clinical investigation. These approaches aim to overcome immune effector-cell exhaustion and counteract the glioma-induced immunosuppressive milieu. As reported above, the combined blockade of immune checkpoints showed auspicious signs of activity in GBM mouse models, leading to an increased immune cell infiltration and development of immunologic memory [19,20,21]. Based on all these premises, the evaluation of multiple clinical combinations is ongoing, with anti-PD-1/PD-L1 agents as a standard therapeutic backbone.

A phase I, open-label, multicenter, multi-arm trial studied the safety of the anti- LAG-3 BMS-986016 alone and combined with nivolumab in GBM patients at the first recurrence after standard chemoradiation (NCT02658981). The study also included two urelumab (anti-CD137 mAb) arms closed prematurely due to drug development program discontinuation. Among 33 evaluable patients, the median OS was eight months, with three patients living beyond 20 months [44]. No DLTs were associated with anti-LAG3 monotherapy, while five total DLTs were observed in combination arms of anti-LAG-3 + nivolumab (one grade 3 muscle weakness, two a grade 3/4 edema, one grade 3 hypertension, and one grade 3 syncope) [44]. Another phase I study investigated BLZ945, an oral inhibitor of colony-stimulating factor 1 receptor (CSF1R), alone or in combination with spartalizumab (anti-PD-1) in several solid tumors (NCT02829723). Among evaluable GBM patients (*n* = 18), two PR were reported; among all treated GBM patients (*n* = 24), seven remained on treatment for more than 16 weeks [45]. Phase II of the study enrolling only GBM patients is currently ongoing.

### 3.4. Neoadjuvant Setting

A multi-institutional, randomized, open-label pilot trial evaluated the feasibility and the efficacy of neoadjuvant PD-1 blockade in recurrent, surgically resectable GBM (NCT02337686). A total of 35 patients were randomized to receive pembrolizumab before and after surgery (*n* = 16) or only as adjuvant therapy (*n* = 19). In the neoadjuvant arm of the trial, one course of pembrolizumab was administered approximately two weeks before surgery. Anti-PD-1 therapy was overall well-tolerated, with no unexpected toxicities and no surgical delay in the neoadjuvant arm. Patients receiving pembrolizumab given before surgery showed a significantly better outcome (HR 0.39, 95% CI 0.17–0.94; *p* = 0.04, log-rank test) [46]. Median OS and median PFS were 13.7 months versus 7.5 months and 3.3 months versus 2.4 months for neoadjuvant versus adjuvant pembrolizumab, respectively [46]. Beyond the enthusiasm generated by this significant survival gain, translational data that emerged from this study were of key importance to understand the changes in brain immunity induced by the administration of immunomodulating mAbs. Presurgical PD-1 blockade determined TILs activation leading to an IFN-γ response within the TME, together with an upregulation of T cells and IFN-γ-related gene expression and downregulation of cell cycle-related gene expression within the tumor [46]. Moreover, focal induction of PD-L1 in the TME, enhanced clonal expansion of T cells, decreased PD-1 expression on peripheral blood T cells, and a decreasing monocytic population were observed more frequently in patients treated with neoadjuvant pembrolizumab [46].

A second prospective, single-arm study conducted by Schalper et al. supports a neoadjuvant strategy (NCT02550249). Neoadjuvant administration of nivolumab in 30 GBM patients resulted in enhanced expression of chemokine transcripts, higher immune cell infiltration, and augmented T-cell receptor clonal diversity among TILs, although no significant clinical benefit was noted (median PFS and median OS were 4.1 and 7.3, respectively) [47].

In another exploratory study, 15 patients affected by recurrent and operable GBM received pembrolizumab before and after surgery (NCT02852655). At a median follow-up of 50 months, three patients had a PR, and one had an SD; the median OS was 20 months, with an OS-12 of 63% [48]. Immunophenotyping analysis showed a scarce infiltration of CD4+ and CD8+ T cells and a prevalence of PD-1-positive CD68+ macrophages; no significant increase in the number of CD8+ cytotoxic T cells was found after pembrolizumab treatment [48].

In summary, presurgical PD-1 blockade seems to induce local and systemic immune responses, with enhanced priming and expansion of tumor-specific T cells, probably driven by tumor-associated-antigens exposure and prolonged in subsequent adjuvant doses. These studies provide compelling evidence that neoadjuvant PD-1 inhibition could rescue ICI efficacy in GBM. Well-designed, prospective clinical trials are warranted to assess the real impact of this approach.

## 4. Biomarkers

Unlike other immune-responsive cancers (e.g., melanoma, lung cancer), malignant gliomas are characterized by poor T-cells infiltration, a low tumor mutational burden (TMB), and a strongly immunosuppressive TME, representing the paradigm of what we call a “cold tumor” [54]. As observed in other immune refractory cancers, clinical investigations demonstrated that only a small fraction of GBM patients achieve durable benefit by immune checkpoint modulation. Waiting for effective combinatorial strategies that will hopefully widen the range of responders, there is an urgent need for biomarkers to guide clinicians in patient selection.

Several factors, such as high PD-L1 expression, microsatellite instability (MSI), deficient DNA mismatch repair (MMRd), and a high TMB, among others, have been identified as predictors of responsiveness to checkpoint blockade in different solid malignancies [55]. It is currently unclear whether these biomarkers could also have a predictive significance in the glioma field.

PD-L1 expression widely varies across the different studies in malignant gliomas specimens, ranging from 7.8% to 88%, due to differences in antibodies used for PD-L1 detection, staining pattern (membranous or fibrillary), and cut-off values for PD-L1 expression [56,57]. Notably, among a series of 43 grade II/III and grade IV gliomas, *IDH*-wild-type gliomas appeared as more immune-activated tumors due to a higher rate of TILs infiltration and PD-L1 expression in comparison to *IDH*-mutated gliomas (*p* < 0.001) [58]. A recent metanalysis of nine studies, including 806 GBM patients, indicated that PD-L1 expression in tumor tissues was significantly related to a poor clinical outcome (HR = 1.63, 95% CI: 1.19–2.24, *p* = 0.003) [59]. Beyond the negative prognostic value, neither PD-1 nor PD-L1 expression has been definitively shown to be predictive of response to immunotherapies in gliomas.

In the sole prospective phase III trial published so far, the Checkmate 143 trial, PD-L1 tumor expression was determined retrospectively by a central laboratory on archival tumor tissue from first surgery or recurrence, with PD-L1 positivity defined as membranous staining in ≥1% of tumor cells. Only 26% of tumor specimens in the nivolumab group were PD-L1 positive, and no predictive value was found at a prespecified subgroup analysis [31].

Besides its well-recognized predictive role in GBM patients undergoing standard chemoradiation with TMZ, *MGMT* promoter methylation is emerging as a factor potentially associated with improved outcomes in patients receiving different immunotherapeutic strategies. Again in the Checkmate 143 trial, *MGMT* methylated patients with no baseline corticosteroid use had a trend toward improved OS with nivolumab (17.0 months) vs. bevacizumab (10.1 months) [31]. Moreover, preliminary data from a phase 3 randomized, double-blinded, placebo-controlled clinical trial of an autologous tumor lysate-pulsed dendritic cell vaccine (DCVax^®^-L) for newly diagnosed GBM showed that the median OS was 34.7 months (95% CI 27.0–40.7) and 19.8 months (95% CI 17.9–21.7), in patients with methylated and unmethylated *MGMT*, respectively [60]. A possible explanation for these findings is that *MGMT* methylated tumors exhibit four more times the number of somatic mutations than *MGMT*-unmethylated GBM, making these tumors more immunogenic and more susceptible to the action of different immunotherapeutic approaches [61].

In a retrospective series of 66 adult patients with recurrent GBM treated with PD-1 inhibitors (pembrolizumab or nivolumab), including 17 long-term responders, genomic and transcriptomic analysis revealed a significant enrichment of phosphatase and tensin homolog (*PTEN*) mutations associated with immunosuppressive expression signatures in non-responders (*p* < 0.05), and enrichment of mitogen-activated protein kinase (MAPK) pathway alterations (protein tyrosine phosphatase non-receptor type 11, *BRAF*) in responders (*p* < 0.05). In the same study, correlation analysis showed that *PTEN* mutations are significantly associated with higher levels of macrophages, microglia, and neutrophils in the TME (*p* < 0.05). Of interest, in post-treatment *PTEN* wild-type samples, the density of CD3+ T cells significantly increased compared with pre-treatment samples (*p* = 0.0095), while *PTEN*-mutated samples did not show this pattern [62].

Improved response to checkpoint blockade is associated with a higher TMB across multiple cancer types. This association in HGGs remains uncertain. Fewer than 2% of newly diagnosed GBM and up to 20–30% of recurrent GBM after TMZ exposure exhibit a hypermutated genotype often associated with MMRd. Moreover, lower-grade gliomas treated with TMZ can also recur as hypermutated HGG [63,64]. Previous clinical reports demonstrated the potential activity of ICIs in this setting. Bouffet et al. showed remarkable and durable tumor regressions in two pediatric siblings with recurrent multifocal germline biallelic MMRd GBM treated with nivolumab [65]. In another case, an adult GBM patient diagnosed with a hypermutated genotype in the setting of a polymerase epsilon gene (*POLE*) germline alteration had a sustained response to pembrolizumab therapy after an intracranial and spinal progression [66]. Touat et al. performed a comprehensive molecular analysis in 10,294 gliomas from clinical sequencing panels datasets and a retrospective review of 11 MMRd glioma patients treated with PD-1 blockade. In this study, MMRd gliomas were predominantly associated with TMZ exposure, lacked significant T-cell infiltrates, were not associated with MSI, and were characterized by extensive intratumoral heterogeneity, poor survival, and a low response-rate to immunotherapy [67].

Recently, in two cohorts stratified by TMB, recurrent GBM patients with ≤ median TMB had more prolonged survival after anti-PD-1/PD-L1 blockade than those with > median TMB [68]. Transcriptomic analyses also showed enriched inflammatory gene signatures in recurrent GBM tumors with a low TMB [68]. Survival differences were maintained after excluding *IDH*-mutated, *MGMT* methylated, and hypermutated patients and were not related to steroid dosage [68]. Notably, these associations were not showed in primary GBM patients [68]. Accordingly, in a recent observational study, 13 patients (eight GBM, four anaplastic astrocytomas, one anaplastic oligodendroglioma) with partial or complete loss of mismatch repair proteins had no apparent clinical benefit from pembrolizumab treatment (median PFS 2.2 months, median OS 5.6 months, no partial or complete response) [69]. Likewise, in the phase II study KEYNOTE-158 evaluating pembrolizumab activity in several different non-colorectal cancers harboring MMRd or MSI, only brain tumor patients (*n* = 13) showed no radiological response with a median PFS and OS of 1.1 and 5.6 months, respectively [35].

## 5. Future Perspectives

Despite the strong immunobiological rationale and the compelling preclinical evidence, the failure of large randomized phase III trials of PD-1 blockade both in the adjuvant and recurrent setting may have already certified the end of the clinical experience with immune checkpoint modulation as treatment of malignant gliomas. However, investigators’ impression is that we are yet far from disclosing the full potential of immune-based treatments for HGG patients. One unresolved question is whether the major contributor to these disappointing results is identifiable in the restrictive intracranial environment, once considered an immune-privileged site, with constraints for drug and immune cell access, or in the GBM’s immune-biology itself. We still do not know with certainty if PD-1\PD-L1 blocking antibodies must locate within the tumors to exert their full activity or simply act on peripheral T cells before their central nervous system (CNS) entry. ICIs’ effectiveness against brain metastases from melanoma and lung cancer underscore the relevance of glioma genetics, intratumoral heterogeneity, and multifactorial immunosuppression. The BBB selectively regulating the influx of effector cells and therapeutics, a scarce CD8+ T-cells infiltrate, low APCs populations, and the prevalence of immunosuppressive humoral factors are GBM-specific barriers to immunotherapy efficacy. Moreover, severe lymphopenia and glioma-induced sequestration of T-cells in the bone marrow are commonly observed in GBM patients. Corticosteroids, universally used to alleviate symptoms of vasogenic edema in brain malignancies, have profound immunological consequences. Indeed, steroid therapy decreases intratumoral and systemic levels of T cells and qualitatively impairs lymphocyte functions, limiting PD-1 blockade efficacy [52,54].

However, in all clinical experiences conducted so far, we invariably found a subgroup, albeit small, of patients with a long-lasting clinical benefit from ICI treatment. This observation clarifies the absolute need for a continued investigation to better understand how to counteract glioma-specific T cell dysfunction and immunosuppressive TME and broaden the range of patients achieving such durable tumor control observed in other cancers. Great expectations have been raised by combining ICIs with other potentially synergistic therapeutic approaches. In the absence of data from large prospective trials for such combinations, early-phase studies’ preliminary results appear again not entirely satisfactory. Clinical activity of PD-1/PD-L1 blockade seems not to be significantly increased by the addition of antiangiogenic therapy [36,37,38,39], and the same consideration currently has to be made for dual immune checkpoint blockade. However, a new wave of novel and promising combinatorial strategies of ICIs with different modalities such as oncolytic viruses, vaccines, chimeric antigen receptor (CAR) T cells have begun their clinical development [70], with the hope of effectively boost antitumor immunity and bringing to the clinic effective therapeutic options for HGG patients.

Besides the chapter of combinations currently under clinical investigations, what recently emerged is that the timing of checkpoint inhibition in GBM could represent a crucial factor for success. Results from presurgical or neoadjuvant trials are, in our opinion, exciting and not only for the survival data but mostly for the immunobiological modifications observed after the exposure to PD-1 blockade, skewing TME towards a more immunogenic milieu. Such types of trials, also called “Phase 0, or window of opportunity trials”, provide an in-depth characterization of glioma TME, allowing us to understand the real effects of our pharmacological modulation on the tumor-immune dynamic system into the brain. These novel designs could further lead to new insights into the fundamental immunobiology of the CNS itself and the acquisition of valuable molecular information for intrinsic resistance to ICI treatment. Randomization represents another key component of this novel wave of studies, ensuring the strength of immunologic and transcriptional findings and reducing selection bias. It is imperative to pursue rational clinical trial design and biomarkers discovery to either identify those subsets of patients most likely to experience clinical benefit and avoid discarding potential promising agents due to heterogeneous and unselected populations. As new promising targets continue to emerge and our understanding of brain tumor genomics and immune TME rapidly improves, despite failures and formidable challenges, we firmly believe that the end of immune checkpoints investigation in malignant gliomas has yet to be written.

## Figures and Tables

**Table 1:** Completed and Ongoing ICI Clinical Trials with available data in HGG.

Study Identifier	Study Design	Target Population (*n*)	Intervention	Primary Endpoint(s)	Results	Status	References
**Adjuvant**							
**NCT02017717**	Multicohort phase I/III	Cohort 1c, 1d: ndGBM (*n* = 113)	Nivo + RT +/− TMZ	Safety and tolerability	No new safety signals	Active, not recruiting	[24]
**NCT03174197**	Open label phase I/II	ndGBM (*n* = 60)	Atezo + RT + TMZ	Phase I: safety Phase II: OS	No new safety signals mPFS 10.6 mo mOS 19 mo G3–G4 TRAEs 55%	Active, not recruiting	[25]
**NCT03047473**	Open label phase II	ndGBM (*n* = 24)	Avelumab + RT + TMZ	Safety and tolerability	No new safety signals ORR 50% mPFS 11.9 mo	Active, not recruiting	[26]
**NCT02336165**	Open label multicohort phase II	Cohort A: ndGBM, unmethylated MGMT (*n* = 40)	DUR + RT	OS-12	mOS 15.1 mo G3–G4 TRAEs 35%	Active, not recruiting	[27]
**NCT02617589**	Open label phase III	ndGBM unmethylated MGMT (*n* = 550)	RT + nivo vs RT + TMZ	OS	Primary endpoint not met	Active, not recruiting	[28]
**NCT02667587**	Triple-blind phase III	ndGBM, methylated MGMT (*n* = 693)	RT + TMZ + nivo vs RT + TMZ + placebo	PFS, OS	Primary endpoints not met	Active, not recruiting	[29]
**Recurrent—monotherapies**							
**NCT02017717**	Multicohort phase I/III	Cohort 1, 1b: rGBM (*n* = 40) (NIVO3 = 10; NIVO1 + IPI3 = 10; NIVO3 + IP1 = 20)	Nivo +/− Ipi	Safety and tolerability	No new safety signals NIVO3 better tolerated mOS NIVO3: 10.4 mo NIVO1+IPI3: 9.2 mo NIVO3+IPI1: 7.3 mo	Active, not-recruiting	[30,31]
		Cohort 2 rGBM (*n* = 184 vs. 185)	Nivo vs. BEV	OS	mPFS 1.5 vs. 3.5 mo mOS 9.8 vs. 10 mo ORR 8% vs. 23% mDOR 11.1 vs. 5.3 mo G3–G4 TRAEs 18% vs. 15%		
**NCT02054806**	Multicohort phase Ib	rGBM PD-L1 expression ≥ 1% by IHC (*n* = 26)	Pembro	ORR	ORR 8% mPFS 2.8 mo mOS 13.1 mo G3–G4 TRAEs 19%	Active, not recruiting	[32]
**NCT01375842**	Phase Ia	rGBM (*n* = 16)	Atezolizumab	Safety and tolerability MTD and RP2D	No G3–G4 TRAEs mOS 4.2 mo	Completed	[33]
**NCT02336165**	Open label multicohort phase II	Cohort B: rGBM, BEV-naïve (*n* = 31)	DUR	PFS-6	PFS-6 20% OS-6 59% OS-12 44% G3-4 TRAEs 10%	Active, not recruiting	[34]
**NCT02628067**	Open label multicohort phase II	dMMR/MSI-H recurrent glioma (*n* = 13)	Pembro	ORR	ORR 0% mPFS 1.1 mo mOS 5.6 mo	Recruiting	[35]
**Recurrent—combinations**							
**NCT02336165**	Open label multicohort phase II	Cohort B2-B3: rGBM, BEV-naïve (*n* = 66)	DUR + BEV	PFS-6	Cohort B2 PFS-6: 15.2% G3-4 TRAEs 24% Cohort B3 PFS-6: 21% G3-4 TRAEs 6%	Active, not recruiting	[36,37]
		Cohort C: rGBM, BEV-refractory (*n* = 22)	DUR + BEV	OS-6	36% of pts OS ≥ 22 wks 50% of pts PFS ≥ 8 weeks G3-4 TRAEs 4.5%		
**NCT02337491**	Randomized phase II	rGBM (*n* = 50 vs. 30)	Pembro + BEV vs. Pembro	PFS-6	PFS-6 26% vs. 6.7% mOS 8.8 mo vs. 10.3 mo ORR 20% vs. 0% G3-4 TRAEs 36% vs. 20%	Completed	[38]
**NCT03291314**	Open label phase II	rGBM (*n* = 54) Cohort 1: DEXA ≤1.5 mg Cohort 2: DEXA ≥ 1.5 mg	Avelumab + Axitinib	PFS-6	Cohort 1 PFS-6 22.2% mOS 26.6 wks ORR 33.3% OS-12 22.2% Cohort 2 PFS-6 18.5% mOS 18 wks ORR 22.2% OS-12 11.1%	Completed	[39]
**NCT02313272**	Phase I	rGBM or rAA (*n* = 32)	Pembro + HFSRT + BEV	Safety and tolerability MTD and RP2D	G3-4 TRAEs 34.4% BEVA- naïve pts ORR 83% mDOR 8.44 mo mPFS 7.92 mo mOS 13.45 m OS-12 58.3% BEVA-resistant pts ORR 62% mDOR 5.83 mo mPFS 6.54 mo mOS 9.3 mo OS-12 25%	Active, not recruiting	[40]
**NCT02866747**	Randomized phase I/II	rGBM (*n* = 6, phase I)	Duvalumab + HFSRT	Safety and tolerability OS	No new safety signals	Recruiting	[41]
**NCT02968940**	Open label phase II	IDH-mutated GBM (*n* = 6)	Avelumab + HFSRT	Safety and tolerability PFS-6	No new safety signals mPFS 4.2 mo mOS 10.1 mo	Completed	[42]
**NCT03743662**	Open label phase II	rGBM, methylated MGMT (*n* = 22)	Nivo + Hypo-RT +/− BEV +/− surgery	OS	mPFS 6.7 mo mOS 16 mo PFS-6 50% OS-12 59%	Recruiting	[43]
**NCT02658981**	Phase I	rGBM (*n* = 33)	BMS 986016 (anti-LAG-3 mAb) +/− Nivo	Safety and tolerability MTD	mOS 8 mo 80% of DLTs occurred after cycle 2	Active, not recruiting	[44]
**NCT02829723**	Open label phase I/II	rGBM (*n* = 18)	BLZ945 (CSF-1R inhibitor) +/− spartalizumab (anti-PD-1 mAb)	Safety and tolerability MTD and RP2D	ORR 11% Acceptable safety pattern	Recruiting	[45]
**Neoadjuvant**							
**NCT02337686**	Open label, randomized, phase II	rGBM (*n* = 16 vs. 19)	Neoadjuvant + Adjuvant Pembro vs. Adjuvant Pembro	Immune effector function analysis and correlation with PFS-6	mPFS 3.3 vs. 2.4 mo mOS 13.7 vs. 7.5 mo	Active, not recruiting	[46]
**NCT02550249**	Open label phase II	ndGBM (*n* = 3) rGBM (*n* = 27)	Neoadjuvant Nivo	Changes in percentage and level of PD-L1	mPFS 4.1 mo mOS 7.3 mo	Completed	[47]
**NCT02852655**	Phase I	rGBM (*n* = 15)	Neoadjuvant Pembro	TIL density Safety and tolerability	mOS 20 mo OS-12 63%	Recruiting	[48]

Atezo, atezolizumab; BEV, bevacizumab; CSF-1R, Colony stimulating factor 1 receptor; DEXA, dexamethasone; dMMR, mismatch repair deficiency; DUR, durvalumab; HGG, high-grade gliomas; HSFRT, Hypofractionated Stereotactic Radiation Therapy; Hypo-RT, Hypofractionated Radiotherapy; ICI, immune checkpoint inhibitors; IDH, isocitrate dehydrogenase; IHC, immunohistochemistry; Programmed death-ligand 1; Ipi, ipilimumab; LAG-3, Lymphocyte-activation gene 3; mAb, monoclonal antibody; mDOR, median duration of response; MGMT, O6-methylguanine-DNA methyltransferase; mOS, median overall survival; mPFS, median progression-free survival; MSI-H, high microsatellite instability; MTD, maximum tolerated dose; ndGBM, newly diagnosed glioblastoma; Nivo, nivolumab; NIVO1 + IPI3, nivolumab 1 mg/kg + ipilimumab 3 mg/kg every Q3W for 4 doses followed by nivolumab 3 mg/kg Q2W thereafter; NIVO3, nivolumab 3 mg/kg every Q2W; NIVO3 + IPI1, nivolumab 3 mg/kg + ipilimumab 1 mg/kg Q3W for 4 doses followed by nivolumab 3 mg/kg Q2W thereafter; ORR, overall response rate; OS, overall survival; OS-12, overall survival at 12 months; OS-6, overall survival at 6 months; PD-1, Programmed Death-1; PD-L1, Programmed death-ligand 1; Pembro, pembrolizumab; PFS-6, progression-free survival at 6 months; rAA, recurrent anaplastic astrocytoma; rGBM, recurrent glioblastoma; RP2D, Recommended Phase 2 Dose; RT, radiotherapy; TIL, tumor-infiltrating lymphocytes; TMZ, temozolomide; TRAE, treatment-related adverse events.

## Data Availability

No new data were created or analyzed. Data sharing is not applicable to this article.

## References

[1] Stupp R., Hegi M.E., Mason W.P., Bent M.J.V.D., Taphoorn M.J.B., Janzer R.C., Ludwin S.K., Allgeier A., Fisher B., Belanger K. (2009). Effects of radiotherapy with concomitant and adjuvant temozolomide versus radiotherapy alone on survival in glioblastoma in a randomised phase III study: 5-year analysis of the EORTC-NCIC trial. Lancet Oncol..

[2] Gilbert M.R., Wang M., Aldape K.D., Stupp R., Hegi M.E., Jaeckle K.A., Armstrong T.S., Wefel J.S., Won M., Blumenthal D.T. (2013). Dose-Dense Temozolomide for Newly Diagnosed Glioblastoma: A Randomized Phase III Clinical Trial. J. Clin. Oncol..

[3] Mao H., Lebrun D.G., Yang J., Zhu V.F., Li M. (2012). Deregulated Signaling Pathways in Glioblastoma Multiforme: Molecular Mechanisms and Therapeutic Targets. Cancer Investig..

[4] Chinot O.L., Wick W., Mason W., Henriksson R., Saran F., Nishikawa R., Carpentier A.F., Hoang-Xuan K., Kavan P., Cernea D. (2014). Bevacizumab plus Radiotherapy–Temozolomide for Newly Diagnosed Glioblastoma. N. Engl. J. Med..

[5] Gilbert M.R., Dignam J.J., Armstrong T.S., Wefel J.S., Blumenthal D.T., Vogelbaum M.A., Colman H., Chakravarti A., Pugh S., Won M. (2014). A Randomized Trial of Bevacizumab for Newly Diagnosed Glioblastoma. N. Engl. J. Med..

[6] Tan A.C., Ashley D.M., López G.Y., Malinzak M., Friedman H.S., Khasraw M. (2020). Management of glioblastoma: State of the art and future directions. CA Cancer J. Clin..

[7] Khalil D.N., Smith E.L., Brentjens R.J., Wolchok J.D. (2016). Erratum: The future of cancer treatment: Immunomodulation, CARs and combination immunotherapy. Nat. Rev. Clin. Oncol..

[8] Tomaszewski W., Sanchez-Perez L., Gajewski T.F., Sampson J.H. (2019). Brain Tumor Microenvironment and Host State: Implications for Immunotherapy. Clin. Cancer Res..

[9] Locarno C.V., Simonelli M., Bonecchi R., Locati M., Savino B., Carenza C., Capucetti A., Stanzani E., Lorenzi E., Persico P. (2020). Role of myeloid cells in the immunosuppressive microenvironment in gliomas. Immunobiology.

[10] Weenink B., French P.J., Smitt P.A.S., Debets R., Geurts M. (2020). Immunotherapy in Glioblastoma: Current Shortcomings and Future Perspectives. Cancers.

[11] Topalian S.L. (2017). Targeting Immune Checkpoints in Cancer Therapy. JAMA.

[12] Reardon D.A., Gokhale P.C., Klein S.R., Ligon K.L., Rodig S.J., Ramkissoon S.H., Jones K.L., Conway A.S., Liao X., Zhou J. (2016). Glioblastoma Eradication Following Immune Checkpoint Blockade in an Orthotopic, Immunocompetent Model. Cancer Immunol. Res..

[13] Robert C. (2020). A decade of immune-checkpoint inhibitors in cancer therapy. Nat. Commun..

[14] Soffietti R., Ahluwalia M., Lin N., Rudà R. (2020). Management of brain metastases according to molecular subtypes. Nat. Rev. Neurol..

[15] Fecci P.E., Ochiai H., Mitchell D.A., Grossi P.M., Sweeney A.E., Archer G.E., Cummings T., Allison J.P., Bigner D.D., Sampson J.H. (2007). Systemic CTLA-4 Blockade Ameliorates Glioma-Induced Changes to the CD4+ T Cell Compartment without Affecting Regulatory T-Cell Function. Clin. Cancer Res..

[16] Zeng J., See A.P., Phallen J., Jackson C.M., Belcaid Z., Ruzevick J., Durham N., Meyer C., Harris T.J., Albesiano E. (2013). Anti-PD-1 Blockade and Stereotactic Radiation Produce Long-Term Survival in Mice with Intracranial Gliomas. Int. J. Radiat. Oncol..

[17] Dai B., Qi N., Li J., Zhang G. (2018). Temozolomide combined with PD-1 Antibody therapy for mouse orthotopic glioma model. Biochem. Biophys. Res. Commun..

[18] Park J., Kim C.G., Shim J.-K., Kim J.H., Lee H., Lee J.E., Kim M.H., Haam K., Jung I., Park S.-H. (2019). Effect of combined anti-PD-1 and temozolomide therapy in glioblastoma. OncoImmunology.

[19] Wainwright D.A., Chang A.L., Dey M., Balyasnikova I.V., Kim C.K., Tobias A., Cheng Y., Kim J.W., Qiao J., Zhang L. (2014). Durable Therapeutic Efficacy Utilizing Combinatorial Blockade against IDO, CTLA-4, and PD-L1 in Mice with Brain Tumors. Clin. Cancer Res..

[20] Kim J.E., Patel M.A., Mangraviti A., Kim E.S., Theodros D., Velarde E., Liu A., Sankey E.W., Tam A., Xu H. (2017). Combination Therapy with Anti-PD-1, Anti-TIM-3, and Focal Radiation Results in Regression of Murine Gliomas. Clin. Cancer Res..

[21] Hung A.L., Maxwell R., Theodros D., Belcaid Z., Mathios D., Luksik A.S., Kim E., Wu A., Xia Y., Garzon-Muvdi T. (2018). TIGIT and PD-1 dual checkpoint blockade enhances antitumor immunity and survival in GBM. OncoImmunology.

[22] Khasraw M., Reardon D.A., Weller M., Sampson J.H. (2020). PD-1 Inhibitors: Do they have a Future in the Treatment of Glioblastoma?. Clin. Cancer Res..

[23] Majc B., Novak M., Jerala N., Jewett A., Breznik B. (2021). Immunotherapy of Glioblastoma: Current Strategies and Challenges in Tumor Model Development. Cells.

[24] Omuro A., Vlahovic G., Baehring J., Butowski N., Reardon D., Cloughesy T., Sahebjam S., Lim M., Zwirtes R., Sampson J. (2017). OS07.3 Nivolumab in Combination with Radiotherapy with or without Temozolomide in Patients with Newly Diagnosed Glioblastoma: Updated Results from CheckMate 143. Neuro-Oncology.

[25] Weathers S.-P.S., Kamiya-Matsuoka C., Harrison R.A., Liu D.D., Dervin S., Yun C., Loghin M.E., Penas-Prado M., Majd N., Yung W.K.A. (2020). Phase I/II study to evaluate the safety and clinical efficacy of atezolizumab (atezo; aPDL1) in combination with temozolomide (TMZ) and radiation in patients with newly diagnosed glioblastoma (GBM). J. Clin. Oncol..

[26] Jacques F.H., Nicholas G., Lorimer I., Sikatifoko V., Prévost J. (2019). Avelumab in newly diagnosed glioblastoma multiforme: The SEJ study. J. Clin. Oncol..

[27] Reardon D.A., Kaley T.J., Dietrich J., Clarke J.L., Dunn G., Lim M., Cloughesy T.F., Gan H.K., Park A.J., Schwarzenberger P. (2019). Phase II study to evaluate safety and efficacy of MEDI4736 (durvalumab) + radiotherapy in patients with newly diagnosed unmethylated MGMT glioblastoma (new unmeth GBM). J. Clin. Oncol..

[28] Bristol-Myers Squibb Announces Phase 3 CheckMate -498 Study Did Not Meet Primary Endpoint of Overall Survival with Opdivo (Nivolumab) Plus Radiation in Patients with Newly Diagnosed MGMT-Unmethylated Glioblastoma Multiforme. https://news.bms.com/news/details/2019/Bristol-Myers-Squibb-Announces-Phase-3-CheckMate--498-Study-Did-Not-Meet-Primary-Endpoint-of-Overall-Survival-with-Opdivo-nivolumab-Plus-Radiation-in-Patients-with-Newly-Diagnosed-MGMT-Unmethylated-Glioblastoma-Multiforme/default.aspx.

[29] Bristol Myers Squibb Announces Update on Phase 3 CheckMate -548 Trial Evaluating Patients with Newly Diagnosed MGMT-Methylated Glioblastoma Multiforme. https://news.bms.com/news/details/2020/Bristol-Myers-Squibb-Announces-Update-on-Phase-3-CheckMate--548-Trial-Evaluating-Patients-with-Newly-Diagnosed-MGMT-Methylated-Glioblastoma-Multiforme/default.aspx.

[30] Omuro A., Vlahovic G., Lim M., Sahebjam S., Baehring J., Cloughesy T., Voloschin A., Ramkissoon S.H., Ligon K.L., Latek R. (2018). Nivolumab with or without ipilimumab in patients with recurrent glioblastoma: Results from exploratory phase I cohorts of CheckMate 143. Neuro-Oncology.

[31] Reardon D.A., Brandes A.A., Omuro A., Mulholland P., Lim M., Wick A., Baehring J., Ahluwalia M.S., Roth P., Bähr O. (2020). Effect of Nivolumab vs. Bevacizumab in Patients with Recurrent Glioblastoma: The CheckMate 143 Phase 3 Randomized Clinical Trial. JAMA Oncol..

[32] Reardon D.A., Kim T.M., Frenel J., Simonelli M., Lopez J., Subramaniam D.S., Siu L.L., Wang H., Krishnan S., Stein K. (2021). Treatment with pembrolizumab in programmed death ligand 1–positive recurrent glioblastoma: Results from the multicohort phase 1 KEYNOTE-028 trial. Cancer.

[33] Lukas R.V., Rodon J., Becker K., Wong E.T., Shih K., Touat M., Fassò M., Osborne S., Molinero L., O’Hear C. (2018). Clinical activity and safety of atezolizumab in patients with recurrent glioblastoma. J. Neuro-Oncol..

[34] Reardon D.A., Kaley T.J., Dietrich J., Clarke J.L., Dunn G.P., Lim M., Cloughesy T.F., Gan H.K., Park A.J., Schwarzenberger P. (2017). Phase 2 study to evaluate safety and efficacy of MEDI4736 (durvalumab [DUR]) in glioblastoma (GBM) patients: An update. J. Clin. Oncol..

[35] Marabelle A., Le D.T., Ascierto P.A., Di Giacomo A.M., De Jesus-Acosta A., Delord J.-P., Geva R., Gottfried M., Penel N., Hansen A.R. (2020). Efficacy of Pembrolizumab in Patients with Noncolorectal High Microsatellite Instability/Mismatch Repair–Deficient Cancer: Results From the Phase II KEYNOTE-158 Study. J. Clin. Oncol..

[36] Reardon D., Kaley T., Dietrich J., Clarke J., Dunn G., Lim M., Cloughesy T., Gan H., Park A., Schwarzenberger P. (2018). Atim-38. Phase 2 study to evaluate the clinical efficacy and safety of medi4736 (durvalumab, durva) + bevacizumab (bev) in bev-naïve patients with recurrent glioblastoma (gbm). Neuro-Oncology.

[37] Reardon D., Kaley T., Dietrich J., Clarke J.L., Dunn G.P., Lim M., Cloughesy T., Gan H.K., Park A., Schwarzenberger P. (2017). Atim-12. Phase 2 study to evaluate the clinical efficacy and safety of medi4736 (durvalumab [dur]) in patients with bevacizumab (bev)-refractory recurrent glioblastoma (gbm). Neuro-Oncology.

[38] Nayak L., Molinaro A.M., Peters K.B., Clarke J.L., Jordan J.T., de Groot J.F., Nghiemphu P.L., Kaley T.J., Colman H., McCluskey C. (2021). Randomized Phase II and Biomarker Study of Pembrolizumab plus Bevacizumab versus Pembrolizumab Alone for Patients with Recurrent Glioblastoma. Clin. Cancer Res..

[39] Awada G., Ben Salama L., De Cremer J., Schwarze J.K., Fischbuch L., Seynaeve L., Du Four S., Vanbinst A.-M., Michotte A., Everaert H. (2020). Axitinib plus avelumab in the treatment of recurrent glioblastoma: A stratified, open-label, single-center phase 2 clinical trial (GliAvAx). J. Immunother. Cancer.

[40] Sahebjam S., A Forsyth P., Tran N.D., Arrington J.A., Macaulay R., Etame A.B., Walko C.M., Boyle T., Peguero E.N., Jaglal M. (2020). Hypofractionated stereotactic re-irradiation with pembrolizumab and bevacizumab in patients with recurrent high-grade gliomas: Results from a phase I study. Neuro-Oncology.

[41] Pouessel D., Mervoyer A., Larrieu-Ciron D., Cabarrou B., Robert M., Frenel J., Olivier P., Mounier M., Moyal E.C.-J. (2019). OS4.4 Hypofractionated stereotactic radiotherapy and anti-PDL1 Durvalumab combination in recurrent glioblastoma: Results of the phase I part of the phase I/II STERIMGLI trial. Neuro-Oncology.

[42] Kurz S., Silverman J.S., Hochman T., Nayak L., Arrillaga-Romany I., Lee E., Patel A., Delara M., Hsu F., Imtiaz T. (2019). Atim-37. Phase II, open-label, single arm, multicenter study of avelumab with hypofractionated radiation (hfrt) for adult patients with secondarily transformed IDH-mutant glioblastoma (gbm). Neuro-Oncology.

[43] Grommes C., Mehta M., Miller A., Daras M., Piotrowski A., Boire A., Butler B., Stone J., Pentsova E., Ko-techa R. CTIM-15-Preliminary results of a phase II study of nivolumab with hypofractionated re-radiation and bevacizumab for recurrent MGMT methylated glioblastoma. Presented at the SNO Virtual Meeting.

[44] Lim M., Ye X., Piotrowski A.F., Desai A.S., Ahluwalia M.S., Walbert T., Fisher J.D., Desideri S., Nabors L.B., Wen P.Y. (2020). Updated safety phase I trial of anti-LAG-3 alone and in combination with anti-PD-1 in patients with recurrent GBM. J. Clin. Oncol..

[45] Lin C.-C., Gil-Martin M., Bauer T.M., Naing A., Lim D.W.-T., Sarantopoulos J., Geva R., Ando Y., Fan L., Choudhury S. Abstract CT171: Phase I Study of BLZ945 Alone and with Spartalizumab (PDR001) in Patients (Pts) with Advanced Solid Tumors. Proceedings of the Tumor Biology, American Association for Cancer Research.

[46] Cloughesy T.F., Mochizuki A.Y., Orpilla J.R., Hugo W., Lee A.H., Davidson T.B., Wang A.C., Ellingson B.M., Rytlewski J.A., Sanders C.M. (2019). Neoadjuvant anti-PD-1 immunotherapy promotes a survival benefit with intratumoral and systemic immune responses in recurrent glioblastoma. Nat. Med..

[47] Schalper K.A., Rodriguez-Ruiz M.E., Diez-Valle R., López-Janeiro A., Porciuncula A., Idoate M.A., Inogés S., De Andrea C., De Cerio A.L.-D., Tejada S. (2019). Neoadjuvant nivolumab modifies the tumor immune microenvironment in resectable glioblastoma. Nat. Med..

[48] De Groot J., Penas-Prado M., Alfaro-Munoz K., Hunter K., Pei B.L., O’Brien B., Weathers S.-P., Loghin M., Matsouka C.K., Yung W.K.A. (2019). Window-of-opportunity clinical trial of pembrolizumab in patients with recurrent glioblastoma reveals predominance of immune-suppressive macrophages. Neuro-Oncology.

[49] Lee W.S., Yang H., Chon H.J., Kim C. (2020). Combination of anti-angiogenic therapy and immune checkpoint blockade normalizes vascular-immune crosstalk to potentiate cancer immunity. Exp. Mol. Med..

[50] Dunn-Pirio A.M., Vlahovic G. (2017). Immunotherapy approaches in the treatment of malignant brain tumors. Cancer.

[51] Diaz R.J., Ali S., Qadir M.G., De La Fuente M.I., Ivan M.E., Komotar R.J. (2017). The role of bevacizumab in the treatment of glioblastoma. J. Neuro-Oncology.

[52] Iorgulescu J.B., Gokhale P.C., Speranza M.C., Eschle B.K., Poitras M.J., Wilkens M.K., Soroko K.M., Chhoeu C., Knott A., Gao Y. (2021). Concurrent Dexamethasone Limits the Clinical Benefit of Immune Checkpoint Blockade in Glioblastoma. Clin. Cancer Res..

[53] Mann J., Ramakrishna R., Magge R., Wernicke A.G. (2018). Advances in Radiotherapy for Glioblastoma. Front. Neurol..

[54] Jackson C.M., Choi J., Lim M. (2019). Mechanisms of immunotherapy resistance: Lessons from glioblastoma. Nat. Immunol..

[55] Havel J.J., Chowell D., Chan T.A. (2019). The evolving landscape of biomarkers for checkpoint inhibitor immunotherapy. Nat. Rev. Cancer.

[56] Berghoff A.S., Kiesel B., Widhalm G., Rajky O., Ricken G., Wöhrer A., Dieckmann K., Filipits M., Brandstetter A., Weller M. (2015). Programmed death ligand 1 expression and tumor-infiltrating lymphocytes in glioblastoma. Neuro-Oncology.

[57] Garber S.T., Hashimoto Y., Weathers S.-P., Xiu J., Gatalica Z., Verhaak R.G.W., Zhou S., Fuller G.N., Khasraw M., De Groot J. (2016). Immune checkpoint blockade as a potential therapeutic target: Surveying CNS malignancies. Neuro-Oncology.

[58] Berghoff A.S., Kiesel B., Widhalm G., Wilhelm D., Rajky O., Kurscheid S., Kresl P., Wöhrer A., Marosi C., Hegi M.E. (2017). Correlation of immune phenotype with IDH mutation in diffuse glioma. Neuro-Oncology.

[59] Hao C., Chen G., Zhao H., Li Y., Chen J., Zhang H., Li S., Zhao Y., Chen F., Li W. (2020). PD-L1 Expression in Glioblastoma, the Clinical and Prognostic Significance: A Systematic Literature Review and Meta-Analysis. Front. Oncol..

[60] Liau L.M., Ashkan K., Tran D.D., Campian J.L., Trusheim J.E., Cobbs C.S., Heth J.A., Salacz M., Taylor S., D’Andre S.D. (2018). First results on survival from a large Phase 3 clinical trial of an autologous dendritic cell vaccine in newly diagnosed glioblastoma. J. Transl. Med..

[61] McDonald K.L., Tabone T., Nowak A.K., Erber W.N. (2015). Somatic mutations in glioblastoma are associated with methylguanine-DNA methyltransferase methylation. Oncol. Lett..

[62] Zhao J., Chen A.X., Gartrell R.D., Silverman A.M., Aparicio L., Chu T., Bordbar D., Shan D., Samanamud J., Mahajan A. (2019). Immune and genomic correlates of response to anti-PD-1 immunotherapy in glioblastoma. Nat. Med..

[63] Hodges T.R., Ott M., Xiu J., Gatalica Z., Swensen J., Zhou S., Huse J.T., De Groot J., Li S., Overwijk W.W. (2017). Mutational burden, immune checkpoint expression, and mismatch repair in glioma: Implications for immune checkpoint immunotherapy. Neuro-Oncology.

[64] Wang J., Cazzato E., Ladewig E., Frattini V., Rosenbloom D.I.S., Zairis S., Abate F., Liu Z., Elliott O., Shin Y.-J. (2016). Clonal evolution of glioblastoma under therapy. Nat. Genet..

[65] Bouffet E., Larouche V., Campbell B.B., Merico D., De Borja R., Aronson M., Durno C., Krueger J., Cabric V., Ramaswamy V. (2016). Immune Checkpoint Inhibition for Hypermutant Glioblastoma Multiforme Resulting From Germline Biallelic Mismatch Repair Deficiency. J. Clin. Oncol..

[66] Johanns T.M., Miller C.A., Dorward I.G., Tsien C., Chang E., Perry A., Uppaluri R., Ferguson C., Schmidt R.E., Dahiya S. (2016). Immunogenomics of Hypermutated Glioblastoma: A Patient with Germline POLE Deficiency Treated with Checkpoint Blockade Immunotherapy. Cancer Discov..

[67] Touat M., Li Y.Y., Boynton A.N., Spurr L.F., Iorgulescu J.B., Bohrson C.L., Cortes-Ciriano I., Birzu C., Geduldig J.E., Pelton K. (2020). Mechanisms and therapeutic implications of hypermutation in gliomas. Nat. Cell Biol..

[68] Gromeier M., Brown M.C., Zhang G., Lin X., Chen Y., Wei Z., Beaubier N., Yan H., E Herndon J., Desjardins A. (2021). Very low mutation burden is a feature of inflamed recurrent glioblastomas responsive to cancer immunotherapy. Nat. Commun..

[69] Lombardi G., Barresi V., Indraccolo S., Simbolo M., Fassan M., Mandruzzato S., Simonelli M., Caccese M., Pizzi M., Fassina A. (2020). Pembrolizumab Activity in Recurrent High-Grade Gliomas with Partial or Complete Loss of Mismatch Repair Protein Expression: A Monocentric, Observational and Prospective Pilot Study. Cancers.

[70] Daubon T., Hemadou A., Garmendia I.R., Saleh M. (2020). Glioblastoma Immune Landscape and the Potential of New Immunotherapies. Front. Immunol..

